# The Adaptive Complexity of Cancer

**DOI:** 10.1155/2018/5837235

**Published:** 2018-12-05

**Authors:** Youcef Derbal

**Affiliations:** Ted Rogers School of Information Technology Management, Ryerson University, Toronto, Canada

## Abstract

Cancer treatment options are expanding to the benefit of significant segments of patients. However, their therapeutic power is not equally realized for all cancer patients due to drug toxicity and disease resistance. Overcoming these therapeutic challenges would require a better understanding of the adaptive survival mechanisms of cancer. In this respect, an integrated view of the disease as a complex adaptive system is proposed as a framework to explain the dynamic coupling between the various drivers underlying tumor growth and cancer resistance to therapy. In light of this system view of cancer, the immune system is in principal the most appropriate and naturally available therapeutic instrument that can thwart the adaptive survival mechanisms of cancer. In this respect, new cancer therapies should aim at restoring immunosurveillance by priming the induction of an effective immune response through a judicious targeting of immunosuppression, inflammation, and the tumor nutritional lifeline extended by the tumor microenvironment.

## 1. Background

Cancer death rates continue to decline while the number of treatment options is increasing for a significant fraction of patients. However, these trends do not apply uniformly to all cancer patients [1–3]. The repertoire of treatment options available to cancer patients, which has traditionally been comprised of surgery, radiation therapy, and chemotherapy, has expanded to include immunotherapy, targeted therapy, and tailored precision therapies based on genetic markers of the disease. While clinical outcomes of these cancer therapies vary across cancer types and patients, drug toxicity, cancer resistance, and recurrence are common challenges for most patients treated with existing therapeutic modalities. The toxicity and side effects of chemotherapy and radiation therapy, which are often followed by cancer recurrence and resistance, are serious limitations to the curative potential of these therapeutic options. On the other hand, targeted therapies based on the selective inhibition of oncogene products have not yielded curative benefits that are commensurate with the anticipated magnitude [4, 5]. The challenges to realizing the therapeutic potential of targeted therapies are rooted in the evolving genetic diversity of cancer cells (CCs) and the rewiring of oncogenic pathways in response to treatment. Immunotherapy is another therapeutic modality ushered with much optimism [6], but whose curative potential has not been translated into concrete clinical benefits to the majority of patients due to cancer resistance to therapy [7]. Meanwhile combination therapies are being used to enhance clinical response and reduce therapeutic resistance [8–10]. However, harnessing the full potential of these cancer therapies requires more rational designs of drug combinations [11–13].

Cancer research is yielding an ever increasing body of knowledge about the biology of cancer and its hallmarks [14, 15]. This has translated into advances in clinical treatments of cancer with an expanding spectrum of treatment options available to patients. Despite these significant advances, cancer resistance to therapy, recurrence, and metastasis continue to pose a formidable barrier to a cure for all patients [7, 16–22]. Underlying these barriers is the continuing challenge to develop effective cancer treatments that can adequately account for the complex dynamics of cancer that emerge from the effects and nonlinear coupling of the evolving genetic diversity of cancer cells, the evolving cellular heterogeneity of the tumor, the response of the immune system, the metabolic reprogramming of cancer cells, and the tumor promoting role of the immunosuppressive and inflammatory state of the tumor microenvironment (TME). However, it is worth noting that while the system dynamics of the disease are not only determined by genetic alterations but also dependent on other dimensions, including immune response and the TME, there may have been a disproportionate focus on genomics guided explorations of cancer treatments. Although such focus may be warranted given the genetic basis of cancer, seeing the disease through the singular lens of its underlying genetic drivers overlooks the tumor promoting processes emerging from the dynamic interactions between cancer cells and the TME. In this respect, this review explores the nature of cancer as a complex adaptive system of causal effects and feedbacks linking the actions of dysregulated oncogenic pathways, the metabolic flexibility of cancer cells, the immunosuppressive and inflammatory state of the TME, and the response of the immune system to tumor growth dynamics. This approach to the understanding of cancer dynamics opens the door for the application of various mathematical and computational methods, from the field of complex adaptive systems [23, 24], to the study of cancer and the exploration of more effective therapeutic strategies. Ultimately, computational models reflecting the understanding of cancer as a complex adaptive system (CAS) may inform the development of combination therapies, which hold a promising potential in the fight against cancer. Indeed, clinically validated CAS mathematical/computational models of the coupled tumor promoting processes may embody a more faithful consideration of cancer complexity and may as a result be more pertinent to the selection of combination drugs with potential drug synergies or additive effects.

## 2. Metabolic Flexibility of Cancer

**Figure 1:**
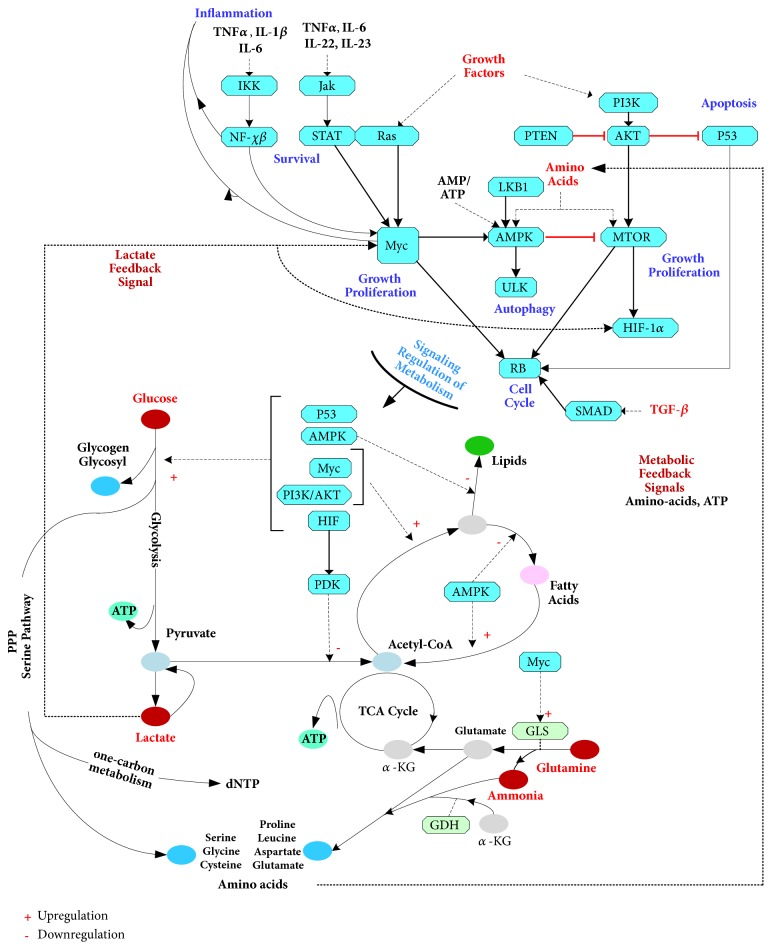
The reprogramming of metabolism in cancer involves positive feedback loops with metabolic outputs such as amino-acids, lactate, and ATP serving as stimuli of the signaling pathways, which are in turn driving the upstream regulators of metabolic enzymes [25–43]. PPP, pentose phosphate pathway; ATP, adenosine triphosphate; AMP, adenosine monophosphate; dNTP, deoxynucleotide; *α*-KG, *α*-ketoglutarate.

The emergence of malignant tumors from healthy tissue and their persistent growth and proliferation suggests that the metabolic states of cancer cells are robustly confined to trajectories that privilege the upregulation of cell growth and division. This entails the need for an incessant sourcing of nutrients and growth factors from the TME to maintain the signaling and metabolic circuitry operating within a regime that promotes growth and rapid cell division. The robust locking of cancer dynamics in a state of growth and proliferation is induced by a closed loop system between the dysregulated mitogenic pathways and their downstream glycolysis and oxidative phosphorylation (OXPHOS) metabolic pathways (see Figure 1). Driven by growth factors and cytokines supplied by cancer associated fibroblast (CAFs) and tumor associated macrophages (TAMs) [70], the positive feedback signals linking the metabolic outputs of dysregulated glycolysis and OXPHOS and the upstream altered mitogenic pathways are speculated to be the primary enablers of the sustained runaway growth and proliferation of cancer cells. The robust dynamics induced by these positive feedback loops drive tumor growth and the syphoning of nutrients such as glucose, glutamine, arginine, and tryptophan from the TME [65]. Consequently, the TME accumulates higher concentrations of metabolic byproducts resulting from tumor growth and necrosis, including lactate, ammonia, glutamate, adenosine, potassium, and prostaglandins [44, 71, 72]. While many of these metabolites were previously considered as waste byproducts of cancer metabolism, mounting evidence suggests that these are recycled by cancer cells to feed their appetite for nutrients and growth promoting signals. In particular, lactate is used by cancer cells as an inflammatory mediator driving growth through MYC, itself shown to be programming inflammation [25, 73, 74]. Furthermore, lactate is recycled by oxidative cancer cells to feed OXPHOS and promotes the uptake of glutamine [26]. On the other hand, the recycling of ammonia has been shown to upregulate amino-acid synthesis in breast cancer, where it is used as a source of nitrogen in the synthesis of glutamate, aspartate, and proline [27, 71].

The increased concentrations in the TME of cytokines and growth factors such as CCL2, IL-1*β*, M-CSF, TGF-*β*, and VEGF, induced by cancer cells and the intervention of the immune system, promote the enlistment of CAFs and TAMs as contributors of nutrients and growth factors needed by cancer cells. This leads to the emergence of a system of mutual dependence between CAFs, TAMs, and cancer cells comprised of multiple feedback loops that stimulate tumor growth [70]. The genetic diversity of cancer cells and the nonuniform spatial distribution of oxygen, nutrients, growth factors, and both proinflammatory and anti-inflammatory cytokines in the TME does not preclude the possibility that the nature of metabolic coupling between cancer cells and CAFs may vary from one region of the tumor to the next. In particular, lactate secreted by glycolytic cancer cells is found to be recycled by CAFs to drive OXPHOS [75], leading to oxidative stress-induced autophagy and the subsequent provision of nutrients to neighboring cancer cells [76]. However, the dynamic interactions between cancer cells and CAFs can also be shaped by a metabolic coupling induced by the reverse Warburg effect, whereby cancer cells adopt aerobic metabolism fueled by lactate excreted by CAFs obligated to upregulate glycolysis [77, 78]. The tumor promoting effects of CAFs is further enhanced with the secretion of growth factors and cytokines that serve as positive feedback signals to the mitogenic pathways of cancer cells. Indeed, CAFs have been shown to upregulate the expression of metalloproteinase MMP-2 and MMP-9 in pancreatic cancer [78]. This leads to extracellular matrix (ECM) degradation and the release and activation of TGF-*β* in the TME and its tumor promoting effect through the PI3K and MAPK pathways [79, 80]. The role of CAFs as a source of positive feedback from the TME to the growth and proliferation of cancer cells has also been shown to be mediated through their secretions of VEGF and IL-6, which drive the JAK/STAT pathway [81].

The metabolic flexibility exhibited by cancer cells in their interactions with CAFs is an adaptive response to the changing nutritional and inflammatory states of their adjacent TME regions. The emergence of this metabolic adaptation may be promoted by the evolving genetic diversity of cancer cells which enables the sampling of a large possible set of altered operational configurations of growth and proliferation pathways that are favorable to the reprograming of metabolism. Such reprogramming would ultimately lead cancer cells to converge to metabolic states (oxidative, glycolytic, or intermediate) that support tumor growth. Overall, the stochastic genetic diversity of cancer cells and the tumor metabolic heterogeneity, which are both evolving in a closed loop system with the changing inflammatory and nutritional states of the TME, may constitute the key drivers that underlie the emergence of the complex adaptive dynamics of tumor growth. These complex dynamics are further modulated by the intervention of the immune system which exacerbates the TME inflammatory state. Ultimately, the immunoediting-driven genetic diversity of the tumor, combined with the scarcity of nutrients (amino-acids and glucose) in the TME, leads to the progressive incapacitation of the immune system and eventually to immune escape.

## 3. Metabolic Pathways of Immunosuppression

**Figure 2:**
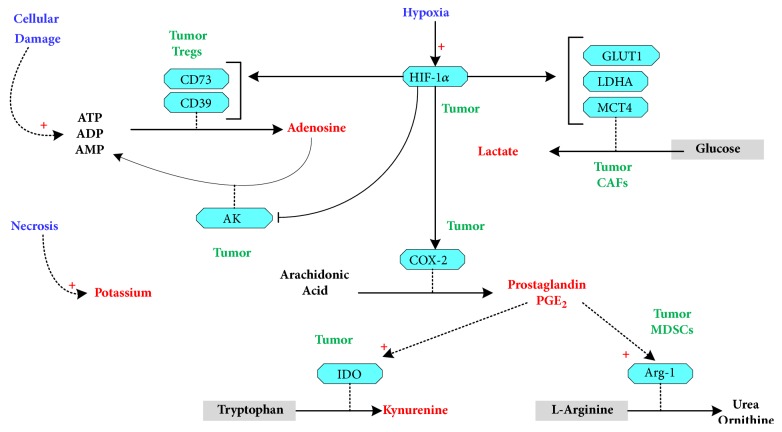
Metabolic pathways of immunosuppression. TME immunosuppression is induced by the elevated concentrations of immunosuppressive metabolites such as lactate, kynurenine, potassium, and adenosine, released in the TME by cancer cells and other TME resident cells [25, 44–53]. ATP, adenosine triphosphate; ADP, adenosine diphosphate; AMP, adenosine monophosphate; MDSCs, myeloid derived suppressor cells; CAFs, cancer associated fibroblast; Tregs, regulatory T cells; myeloid derived suppressor cells; AK, adenosine kinase.

Cancer reprograming of metabolism and its robust confinement to a growth promoting state trajectory, driven by the dysregulated mitogenic pathways and the metabolic coupling between CCs ad CAFs, stabilize tumor growth dynamics. The resulting depletion of oxygen and nutrients in the TME leads to a progressive spread of cellular damage and tumor necrosis. These, in turn, drive cancer metabolism to yield higher concentrations of lactate, kynurenine, potassium, and adenosine in the TME, hence providing feedback signals that maintain the metabolic dysregulation of cancer as well as drive TME immunosuppression (see Figure 2). In particular, hypoxic stress leads to the induction of HIF-1*α* in cancer cells which consequently upregulates CD39/CD73 [44–47] and represses adenosine kinase [82], causing increased accumulation of adenosine in the extracellular space [83]. Furthermore, the highly expressed CD39/CD73 on the surface of T regulatory cells (Tregs) also contributes to the raised TME levels of adenosine [84]. The persistence of higher concentrations of adenosine in the TME limits the inflammatory responses and amplifies immunosuppression through its inhibition of T, T helper cells and cytokine production [44, 47, 84, 85]. Adenosine augments the immunosuppressive effect of lactate which itself is released at higher rate due to the upregulation of glycolysis induced by HIF-1*α* and other end effectors of the dysregulated mitogenic pathways [25]. The progressive necrosis that accompanies tumor growth and hypoxia leads to the accumulation of higher concentrations of potassium ions in the TME, which constitute yet another antitumoricidal effector in the TME [72, 86, 87]. The activation of HIF-1*α* following hypoxic stress also upregulates Cox-2 and leads as a result to higher levels of prostaglandin E2 (PGE2), hence promoting inflammation and cancer proliferation [48–51]. Furthermore, prostaglandins upregulate Indoleamine 2-3 dioxygenase (IDO) which catabolizes tryptophan into another immunosuppressive metabolite, namely, kynurenine [52], and affect the expression of arginase (Arg-1) in tumor cells as well as in myeloid derived suppressor cells (MDSCs) [44, 53]. The regulatory effect of PGE2 on IDO and arginase leads to the depletion of arginine and tryptophan from the TME and consequently depriving T cells of nutrients essentials to their proliferation and activation [44, 88–90]. The limited availability of tryptophan and arginine in the TME, combined with the syphoning of glucose and glutamine by cancer cells, leads to the incapacitation of the tumor infiltrating cytotoxic T cells [65–68]. Furthermore, both the depletion of amino-acids, such as arginine and tryptophan, and the increased concentrations of immunosuppressive molecules in the TME limit the cytotoxic activities of NK cells [44, 91–93]. Overall, abnormal tumor growth is initiated by the genetic dysregulation of mitogenic pathways and stabilized by the TME promoted upregulation of glycolysis, which ultimately leads to hypoxia. The cascading effects of this stable growth and hypoxia reshape the TME into an immunosuppressive and nutrient poor milieu incapacitating T and NK cells while promoting the immunosuppressive actions of Tregs and MDSCs [44, 94]. However, recent evidence suggests that tumor infiltrating immune cells also reprogram their metabolism [95]. While the corresponding mechanistic details and the extent to which such reprogramming contributes to tumor progression are yet to be fully elucidated, this aspect constitutes yet another dimension of cancer complexity that needs to be considered in the search for more effective therapies.

## 4. Immune Response and Inflammation

**Figure 3:**
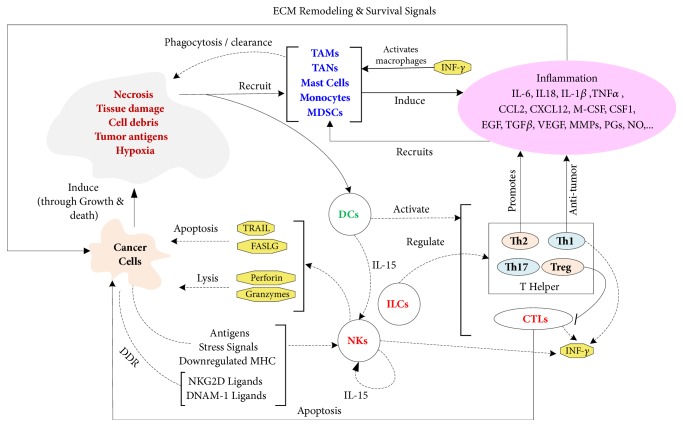
Immune response and inflammation. The immune response to cancer growth is mediated by multiple feedback loops involving innate and adaptive immune cells and the resulting tumor promoting inflammation [54–64]. TAMs, tumor associate macrophages; TANs, tumor associated neutrophils; MDSCs, myeloid derived suppressor cells; DCs, dendritic cells; NKs, natural killer cells; ILCs, Innate lymphoid cells; CTLs, cytotoxic T lymphocytes; MHC, major histocompatibility complex; ECM, extracellular matrix; MMPs, matrix metalloproteinases, PGs, Prostaglandins; NO, Nitric Oxide; DDR, DNA damage response; Th1, T helper 1; Th2, T helper 2; Th17, T helper 17; Treg, T regulatory.

The immune response to an emerging tumor growth is generally understood through the lens of the immunoediting hypothesis, whereby the engagement dynamics of the immune system with the nascent tumor undergoes three progressive phases [96, 97]. In the first phase of engagement, the immune system succeeds in eliminating most cancer cells leading to an equilibrium phase, where sufficient cancer cells are killed, hence preventing the tumor from entering a stage of proliferative growth. However, during this equilibrium stage, the immune system applies its selective pressure on tumor cells which leads to the emergence of less immunogenic clones that escape immune killing and grow thereafter uncontrollably, ushering the escape phase of immunoediting. While the immunoediting hypothesis provides an intuitively simple and evidence supported potential explanation of the immune response to cancer growth, such response may be dynamically coupled to tumor inflammation and autophagy [54–56]. In particular, autophagy helps the anti-tumor immune response by enhancing antigen processing and presentation while quelling tumor inflammation by clearing cellular waste, and dead cancer cells ([55] and references therein). On the other hand, the maintenance of a robust immunogenic response to tumor antigens drives a sustained activation of macrophages by cytokines, such as INF-*γ* and TNF-*α* released by natural killer (NK) cells, innate lymphoid cells (ILCs), and T cells [57–59]. This in turn enhances the recruitment of more neutrophils, macrophages, and MDSCs to the TME, inducing an anti-tumor inflammation that can presumably facilitate the elimination of cancer cells before being resolved through the actions of the feedback system involving immune, phagocytic, and inflammatory cells (see Figure 3). This scenario of successful immune surveillance is promoted by a nonimpaired DNA damage response (DDR) [98–101], a regulated autophagy, an effective presentation of tumor antigens, and a tumoricidal effectiveness of the innate and adaptive immune cells, in particular NK cells and cytotoxic T lymphocytes (CTL). For instance, DDR alerts the immune system through the upregulation of protein ligands that sensitize NK cells and CTLs, via their NKG2D and DNAM-1 receptors, to induce cancer cell lysis [99, 100]. However, cellular senescence associated with persistent DDR leads to raised secretions of inflammatory cytokines by senescent cells [101–103]. Functional dysregulation of any element in the closed loop system tying inflammation and immune response (see Figure 3), such as dysregulated autophagy in monocytes and granulocytes or an immunosuppression induced impotence of the immune cells, would lead to a spiraling and unbreakable cycle of sustained and unresolvable inflammation, which consequently helps the causal levers of immune escape. This interplay between inflammation and immune response presents significant challenges to the exploration of effective therapeutic interventions that can disrupt the tumor promoting dynamics of inflammation while enhancing the cytotoxic activities of the immune system [104–107].

## 5. Metabolic Inhibition of Immune Response

**Figure 4:**
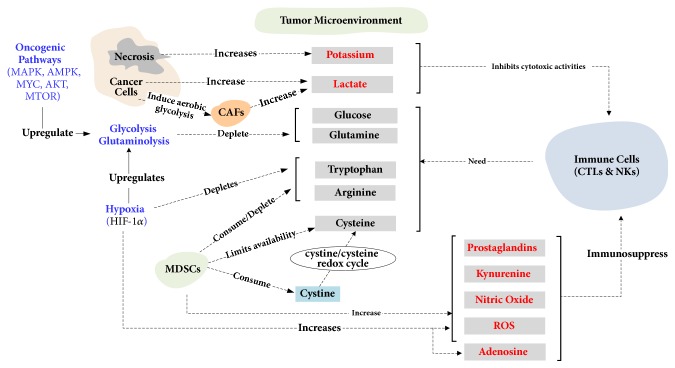
Metabolic inhibition of immune response. Cancer reprograming of metabolism leads to the TME depletion in nutrients such as glucose, glutamine, tryptophan, and arginine [37, 44, 65–68], and the release of immunosuppressive metabolites such as kynurenine, lactate, and prostaglandins [25, 45–53, 69]. ROS, reactive oxygen species; MDSCs, myeloid derived suppressor cells; CAFs, cancer associated fibroblasts; NKs, natural killer cells; CTLs, cytotoxic T lymphocytes.

The insatiable appetite of cancer cells for glucose and glutamine, combined with the upregulated metabolism of arginine and tryptophan, driven by hypoxia, drains the TME of glucose and amino acids required for the activation and proliferation of immune cells. Indeed, it has been suggested that cancer cells act as nutrient sinks in the TME, depriving NK and cytotoxic T cells of oxygen, amino acids, and fatty acids necessary for their proliferation and tumoricidal functions [44, 65]. However, it is also known that T cells and macrophages undergo metabolic reprogramming to meet their bioenergetics needs for rapid proliferation [108, 109]. In particular, glucose depletion induces an AMPK-dependent T cell upregulation of glutaminolysis and glutamine uptake to feed the tricarboxylic acid cycle (TCA), as well as leading to reduced energy consumption due to AMPK inhibition of mTOR–regulated mRNA translation [110, 111]. This metabolic adaptation of T cells parallels the metabolic flexibility of cancer cells in response to nutrient dearth, hence giving support to the notion of a metabolic competition taking place between immune and cancer cells over the scarcity of nutrients in the TME [67, 109]. In such competition, the persistent effects of the oncogenic alterations of MYC, HIF-1*α*, and the RAS/ERK and AKT/PI3K/mTOR pathways, which drive the upregulation of glycolysis and glutaminolysis, may endow cancer cells with a competitive advantage over immune cells, facilitating as a result the incapacitation of the T and NK cells through the depletion of nutrients in the TME [44, 65–68]. Indeed, while metabolic reprograming in cancer cells is driven by long-lasting oncogenic alterations, the upregulation of aerobic glycolysis for T cells is not maintained beyond the duration of the peak immune response [109]. Furthermore, in response to hypoxia, HIF-1*α* upregulates glycolysis and promotes the depletion of tryptophan and arginine leading to an increase in the accumulation of the immunosuppressive prostaglandins and kynurenine in the TME. In addition, the raised levels of lactate, resulting from the upregulation of glycolysis, inhibit the cytotoxic activities of immune cells [112], while hypoxia induces an increase in the levels of adenosine, NO (nitric oxide), and ROS (reactive oxygen species) in the TME, therefore contributing to its immunosuppressive milieu (see Figure 4). In other words, the competition for scarce nutrients is tilted to the advantage of cancer cells which maintain an upregulated glycolysis that syphons nutrients while promoting an immunosuppressive TME that limits the capacity of immune cells to source the necessary nutrients for their proliferation. Furthermore, colluding with cancer cells in their metabolically mediated antagonism to the immune response, MDSCs are also involved in the competition for amino acids needed by immune cells [44, 113] as well as contribute to the immunosuppressive milieu of the TME [114–116].

## 6. Adaptive Complexity of Cancer

**Figure 5:**
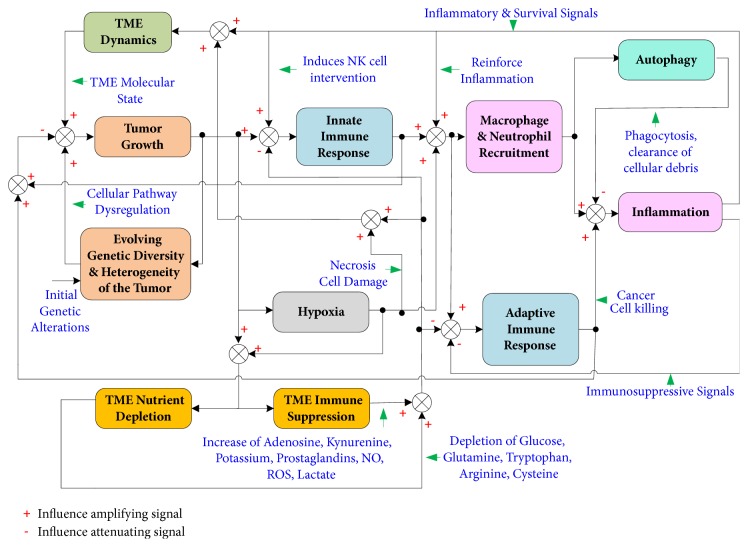
The complex adaptive dynamics of cancer are driven by the multiplicity of causal effects and feedbacks coupling the genetically evolving tumor, the response of the immune system, and the changing metabolic state of the TME.

Although the immunoediting hypothesis explains the potential process through which cancer cells escape immune surveillance, the adaptive TME state feedback has a pivotal role in tumor growth progression. In particular, the emergence of cancer growth driven by dysregulated mitogenic pathways induces a response of the innate immune system along with tissue inflammation, followed by the intervention of the adaptive immune system. In the best case scenario, cancer cells are eliminated, the inflammation is resolved, and tissue homeostasis is reestablished. However, given the multiple feedback loops that modulate the driver signals of tumor growth as well as immune response and inflammation, the potential trajectories of tumor growth dynamics may be infinitely many (see Figure 5). Indeed, the network of interactions coupling cancer dysregulated pathways, metabolic adaptation of cancer cells, immune suppression, metabolic competition, inflammation, immune response, and the evolving genetic diversity and cellular heterogeneity of the tumor, constitutes a nonlinear dynamical system driving tumor growth progression. Such system has the hallmarks of a complex adaptive system whose behavior is not necessarily predictable from the dynamics of its components [117]. For instance, targeting various oncogenic pathways based on their known involvements in different cancer types does not yield a lasting response [118–121]. In fact, acquired resistance to treatment represents a strong manifestation of the adaptive complexity of cancer, defying the curative expectations of therapeutic modalities designed to target well understood cancer drivers.

The adaptive complexity of cancer growth explains the challenges facing the development of therapeutic interventions that can effectively blunt the progression of cancer and avoid the induction of resistance. Indeed, the adaptive dynamics of cellular interactions in the TME take place within the context of an ever expanding genetic diversity and cellular heterogeneity of the tumor and involve interdependent cellular processes which include mitogenic signaling, metabolism, autophagy, inflammation, and immune response. Furthermore, the maintained inflammatory and immunosuppressive milieu of the TME and the competition over nutrients among the cells of the TME give rise to complex metabolic and angiogenic adaptation dynamics which induce tumor growth trajectories that are difficult to predict. The inherent lack of explicit consideration of these adaptive dynamics of cancer in the design of the commonly used therapeutic strategies (chemotherapy, targeted therapy, hormone therapy, immunotherapy, or the combination thereof) may constitute the root cause of the limited curative success of these therapies to a select subset of cancer types and patients [5, 122–124]. Indeed, given the adaptive complexity of cancer (see Figure 5), it is reasonable to speculate that in the absence of an integrated understanding of such complexity even personalized/precision therapeutic approaches driven by omics signatures of patients would fail for most cases.

The adaptation capacity of cancer rests on the dynamic interactions between a genetically heterogeneous tumor and the cells of the TME, including immune, inflammatory, and stroma cells. These interactions can either be collaborative or competitive cellular couplings giving rise to unresolved inflammation, immunoediting, immunosuppression, and nutrient competitions in the TME, with net tumor progression dynamics that favor growth. The effective targeting of these adaptive dynamics of growth may require an objective estimation of the expected tumor progression trajectory as a function of the evolving genetic diversity and changing metabolic state of the tumor, and the dynamic states of inflammation, immunosuppression, and immune response in the TME. Central to such prediction of the progression trajectory of cancer are the dysregulated signaling and metabolic pathways. These pathways shape the interactions between cancer cells and the TME and consequently the tumor growth dynamics. The dysregulation of these pathways translates into a loss of tissue homeostasis, which reflects the altered information flow, bioenergetics, and biosynthesis in cancer cells, and gives rise to cancer lesions. This characterization of cancer in terms of information flow and energy/biomass synthesis is aligned with the notion that information and energy are the fundamental organizing drivers of complexity in living organisms [125]. Indeed, life depends on the DNA sourced information channeled through the cell signaling and transcriptional regulatory circuitry which stitches together programs of gene expressions that maintain the proteomic and enzymatic networks governing cellular processes and fulfill their needs in energy and biomass. In this respect, cancer dynamics were argued to be driven by mutual dependence between the irregular cellular flow of information and the altered biosynthesis of energy and biomass [70]. This information-energy centered view of cancer may serve as a biologically plausible abstraction since it reflects the fundamental functions of the signaling and metabolic pathways, which are the primary mechanistic drivers of the cell life cycle. In particular, such abstraction frames the understanding that cancer lesions are manifestations of loss of tissue homeostasis caused by the genetically driven dysregulation of signaling and metabolic pathways. These dysregulated pathways shape cancer complexity by integrating the feedback of the TME, which reflects the causal effects of hypoxia, metabolic competition, inflammation, immunosuppression, and immune response.

## 7. Metabolic Incapacitation of Immune Cells

The dynamics of cancer progression are also actively shaped by the intervention of the immune system and the feedback exerted by the evolving metabolic, inflammatory, and immunosuppressive conditions of the TME as discussed earlier. Ultimately, the interplay between metabolic reprograming of cancer cells and the mutual interactions between the tumor and the cells of the TME, including TAMs, CAFs, stroma, and immune cells, determine the dynamic molecular state of the TME. In response, the TME reciprocates with feedbacks that power the adaptive survival mechanisms of cancer cells (Figure 5). Furthermore, the molecular state feedback of the TME is dependent on the levels of inflammatory cytokines, the abundance of immunosuppressive and tumor promoting metabolites, and the abundance levels of nutrients including glucose, amino acids, and oxygen. Intriguingly, the inflammation that is initially induced to reestablish tissue homeostasis and counter nascent cancer lesions becomes a source of survival signals driving the NF-*χβ* and Jak/Stat pathways of cancer cells. On the other hand, the closed loop system of causes and effects between the evolving genetic tumor diversity, tumor growth, hypoxia, metabolic reprogramming, and the mutual interactions between the various cells of the tumor milieu leads to a dynamic metabolic state of the TME that promotes cancer growth. Indeed, metabolic analysis of different tumor types has shown differential abundance of lactate, glutamate, and kynurenine in tumors compared to normal tissue [126, 127]. Both lactate and kynurenine act as tumor promoting TME feedback signals. In particular, lactate is used by oxidative cancer cells through the reverse Warburg effect to feed the TCA cycle [31], while contributing to the acidity and immunosuppressive milieu of the TME [69, 128, 129]. On the other hand, not only is the elevated differential abundance of glutamate correlated with an upregulated glutaminolysis feeding the TCA cycle in cancer, but it is also indicative of the recycling of ammonia that feeds amino acid synthesis in cancer cells [27, 130]. Metabolic analyses have also revealed an increased level of kynurenine in tumors compared to normal tissue for different cancer types [126]. Since kynurenine is a byproduct of tryptophan's catabolism [52], not only does its elevated abundance contribute to the immunosuppressive milieu of the TME, but it may also be a proxy for the state of TME depletion in tryptophan, which is needed by T cells [44, 131]. Hence, elevated kynurenine is another TME feedback signal promoting tumor growth by incapacitating the effector actions of immune cells. Furthermore, the frequently elevated differential abundance of F6P and glutamate across many cancers [126] confirms the understanding that cancer cells siphon glucose and glutamine from the TME and consequently deprive immune cells of needed nutrients. Taken together, the molecular state of the TME reflects the actions of cancer cells as metabolic sinks for nutrients and as sources of immunosuppressive signals and hence enacts a two-prongs lever of immune cell incapacitation.

## 8. Can the Adaptive Complexity of Cancer Be Tamed?

**Figure 6:**
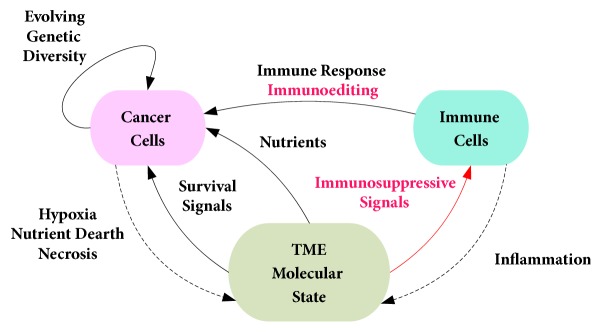
Coarse-grain model of interactions between cancer and immune cells and the effects of the molecular state of the TME.

The evolving genetic diversity of tumors drives the dysregulation of metabolic and signaling circuitry in cancer cells. These dynamics of pathway dysregulations are locked in a closed loop feedback system of causes and nonlinear effects with the metabolic and inflammatory state of the TME and the immunoediting response of the immune system (see Figure 5). Such self-perpetuating closed chain of actions and reactions may explain the limited success of therapeutic interventions that target any singular element in this complex system. Indeed, targeting any component of this closed loop adaptive system, whether it is through kinase inhibition, hormonal therapy, enzyme inhibition, immunotherapy, or any other targeted treatment modality, would induce a reaction of the overall system, which if not accounted for will undermine treatment outcomes. One potentially successful approach to address such disease complexity is to adopt a reductionist and coarse view of cancer as a dual between cancer and immune cells mediated by the molecular state of the TME (see Figure 6). This cancer model has the advantage of representing the complex dynamics of the TME using the corresponding metabolic and inflammatory state variables that are either measureable or amenable to estimation. The reliance on estimated or measured TME molecular state variables enables the reasoning about potential therapeutic levers without discounting the reaction of the entire system of tumor and TME, which would be reflected in the TME state.

The proposed perspective on the adaptive complexity of cancer highlights the metabolic, inflammatory, and immunosuppressive feedback exerted by the TME as a targetable vulnerability of cancer. This proposition is aligned with the increasing interest in therapeutic strategies targeting cancer metabolism [31, 37, 88, 132, 133], inflammation [104, 134–137], and immune suppression [138–140]. However, the potential effectiveness of any therapeutic strategy will depend on the extent to which it will mitigate the adaptive survival and resistance dynamics induced by therapy and the evolving genetic diversity of the tumor subject to interactions with the immune system and other TME cells such as TAMs, and CAFs. This is clearly reflected in the challenges facing the realization of the curative promises of targeted therapies [4, 5]. In this respect, future explorations of new cancer therapies will require a better understanding of the immune response and the molecular state of the TME as modulators of the degree of tissue homeostasis loss caused by the adaptive survival mechanisms of cancer cells. The challenge lies in the fact that the immune response and the TME state feedback are neither linear functions of their effectors, i.e., levels of metabolites, cytokines, and immune cell infiltration in the TME, nor decoupled from the dysregulated cellular processes they affect. In this regard, metabolite and cytokine profiling across different tumor types would be instrumental towards a better understanding of the metabolic, immunosuppressive, and inflammatory barriers preventing an effective immune response. However, tumor growth trajectory and the corresponding evolution of genetic diversity are driven by the dysregulated cellular processes fed by the TME state feedback. In other words, enabling immune intervention by targeting immunosuppression and inflammation would have to be combined with the disruption of the cancer's lifeline extended by the TME state feedback in the form of metabolic fuel, in particular glucose, lactate, and glutamine. Ultimately, cancer therapies should aim at restoring immunosurveillance and preventing the dynamics of interactions between the immune system and cancer from slipping into the immunoediting and escape stages. This proposition is inspired by the fact that the immune system is inherently adaptive in its function and hence is in principal the most appropriate and naturally available therapeutic instrument that can thwart the adaptive survival mechanisms of cancer. However, to induce a curative immune response, therapeutic strategies based on combinations of chemotherapy, radiation, hormone therapy, targeted therapies, and immunotherapy may have to be judiciously developed to progressively drag cancer dynamics away from the growth trajectory to a retreat course where the immune system can fully act within the TME to tame cancer by restoring and maintaining immunosurveillance thereafter. Such therapeutic strategies would be aligned with the “immune normalization” class of therapies which includes B7-H1/PD-1 blockade, whose success is attributed to the resetting of immunity in the TME [141]. Overall, however, the successful development of therapies that can re-enable the immune system in the TME will require a better understanding of the complexity of the TME at least from the vantage points of tumor immunity and the TME molecular state feedback that drives the degree of perturbation of energy/biomass synthesis and cellular signaling/communication underlying sustained tumor growth. In this respect, patient-specific profiling of tumor immunity and the TME molecular state will be needed to provide an objective observation of their effects on tumor growth dynamics (see Figure 6). Such profiling would ultimately support clinical decisions about the type of therapies that can blunt tumor growth and restore immunosurveillance in the TME.

## 9. Conclusions

The causal effects and feedbacks coupling the growth dynamics of genetically evolving and heterogeneous tumors, the changing metabolic and inflammatory state of the TME, the competition over nutrients among the cells of the TME, and the immune response give rise to the adaptive complexity of cancer. This closed loop system induces the emergence of cancer adaptation mechanisms, such as the reprogramming of metabolism and the rewiring of signaling pathways, which guide tumor growth dynamics to trajectories that are privileged by the availability of energy and biomass. This supports the notion that cancer cells apply a two-prong lever of immune cell incapacitation by acting as metabolic sinks for nutrients and as sources of immunosuppressive signals. The inherent lack of explicit consideration of such adaptive complexity of cancer in the commonly used therapeutic strategies may underlie their limited curative success to a select subset of cancer patients. In this respect, the view of cancer as manifestations of tissue loss of homeostasis reflecting the irregularities of information flow and energy/biomass synthesis may constitute an effective abstraction of cancer adaptive complexity that can be leveraged in the exploration of more effective cancer therapeutic strategies.

In light of the complex and adaptive nature of cancer, the immune system is in principal the most appropriate and naturally available therapeutic instrument that can thwart the adaptive survival mechanisms of cancer. In this respect, cancer therapies should assist the antitumoricidal immune response by targeting immunosuppression, inflammation, and the tumor nutritional lifeline (i.e., glucose, glutamine, and lactate) extended by the TME. Ultimately, cancer therapies should aim at restoring immunosurveillance and preventing the dynamics of interactions between the tumor and the immune system from slipping into the immunoediting and escape stages. Indeed, there is a plausible potential that the induction of a curative immune response can be primed through the use of combination therapies that are judiciously designed to progressively steer tumor growth dynamics to trajectories where the immune system can fully act to restore immunosurveillance. However, given the dominant effects of immune cells and the TME in shaping tumor growth dynamics, patient-specific profiling of tumor immunity and the molecular state of the TME will be needed in order to support evidence-based therapeutic decisions.

## Data Availability

No data were used to support this study.
